# Lymphatic vessels: roles and potential therapeutic intervention in rheumatoid arthritis and osteoarthritis

**DOI:** 10.7150/thno.90940

**Published:** 2024-01-01

**Authors:** Siru Zhou, Guangyu Zhao, Ran Chen, Yang Li, Junlan Huang, Liang Kuang, Dali Zhang, Zhijun Li, Haofeng Xu, Wei Xiang, Yangli Xie, Lin Chen, Zhenhong Ni

**Affiliations:** 1 War Trauma Medical Center, State Key Laboratory of Trauma and Chemical Poisoning, Army Medical Center, Daping Hospital, Army Medical University, Chongqing, 40038, People's Republic of China.; 2 Department of Wound Repair and Rehabilitation Medicine, State Key Laboratory of Trauma and Chemical Poisoning, Army Medical Center, Daping Hospital, Army Medical University, Chongqing, 40038, People's Republic of China.; 3 Department of Emergency Surgery, Union Hospital, Tongji Medical College, Huazhong University of Science and Technology, Wuhan, 430022, People's Republic of China.; 4 Rehabilitation Medicine Department, Army Medical Center, Daping Hospital, Army Medical University, Chongqing 400038, People's Republic of China.; 5 The Department of Cardiology, General Hospital of Northern Theater Command, Shenyang 110015, People's Republic of China.

**Keywords:** Rheumatoid arthritis, Osteoarthritis, Inflammatory arthritis, Lymphatic vessels and Drainage function

## Abstract

Lymphatic vessel networks are a main part of the vertebrate cardiovascular system, which participate in various physiological and pathological processes via regulation of fluid transport and immunosurveillance. Targeting lymphatic vessels has become a potent strategy for treating various human diseases. The presence of varying degrees of inflammation in joints of rheumatoid arthritis (RA) and osteoarthritis (OA), characterized by heightened infiltration of inflammatory cells, increased levels of inflammatory factors, and activation of inflammatory signaling pathways, significantly contributes to the disruption of cartilage and bone homeostasis in arthritic conditions. Increasing evidence has demonstrated the pivotal role of lymphatic vessels in maintaining joint homeostasis, with their pathological alterations closely associated with the initiation and progression of inflammatory joint diseases. In this review, we provide a comprehensive overview of the evolving knowledge regarding the structural and functional aspects of lymphatic vessels in the pathogenesis of RA and OA. In addition, we summarized the potential regulatory mechanisms underlying the modulation of lymphatic function in maintaining joint homeostasis during inflammatory conditions, and further discuss the distinctions between RA and OA. Moreover, we describe therapeutic strategies for inflammatory arthritis based on lymphatic vessels, including the promotion of lymphangiogenesis, restoration of proper lymphatic vessel function through anti-inflammatory approaches, enhancement of lymphatic contractility and drainage, and alleviation of congestion within the lymphatic system through the elimination of inflammatory cells. At last, we envisage potential research perspectives and strategies to target lymphatic vessels in treating these inflammatory joint diseases.

## 1. Introduction

The lymphatic vascular network, found in virtually every organ of vertebrates, functions as a unidirectional flow system characterized by low pressure. Under physiological conditions, its primary roles include the elimination of interstitial capillary filtrates and tissue immunosurveillance 1. The significance of lymphatic vessels in a wide range of human diseases is increasingly being acknowledged. Dysfunction of lymphatic vessels is observed in disorders such as pulmonary lymphatic anomalies 2, primary lymphoedema 3, adipose metabolism and obesity 4. Targeting lymphatic vessels has become a potent strategy for treating for cancer 5, neurological disease 6 and facilitating post-myocardial infarction repair 7. The precise regulation of lymphatic vessel structure and function has significant potential for informing the development of innovative therapeutics to a broad range of human diseases.

Inflammatory arthritis, such as rheumatoid arthritis (RA) and osteoarthritis (OA), are characterized by joint pain, swelling, and stiffness 8, 9, which have a high prevalence and is considered as the primary causes of global disability 10. Although the regulation of RA- and OA-associated inflammation may involve distinct mechanisms, the excessive accumulation of catabolic factors, cytokines, and inflammatory cells in joints significantly contributes to the disruption of cartilage and bone homeostasis in these arthritic conditions. The impairment of lymphatic vessels leads to a decline in lymphatic clearance and hyperinflammation, which play crucial roles in the pathogenesis of inflammatory arthritis 11, 12. In this review, we provide a comprehensive overview of the evolving knowledge regarding the structural and functional aspects of the lymphatic vessels in the pathogenesis of RA and OA. In addition, we summarized the potential regulatory mechanisms underlying the modulation of lymphatic function in maintaining joint homeostasis during inflammatory conditions, and further discuss the distinctions between RA and OA. Moreover, we describe therapeutic strategies for inflammatory arthritis based on lymphatic vessels, including the promotion of lymphangiogenesis, restoration of proper lymphatic vessel function through anti-inflammatory approaches, enhancement of lymphatic contractility and drainage, and alleviation of congestion within the lymphatic system through the elimination of inflammatory cells. At last, we envisage potential research perspectives and strategies to target lymphatic vessels in treating these inflammatory joint diseases.

### 1.1 The structure and function of lymphatic vessels

**Figure 1:**
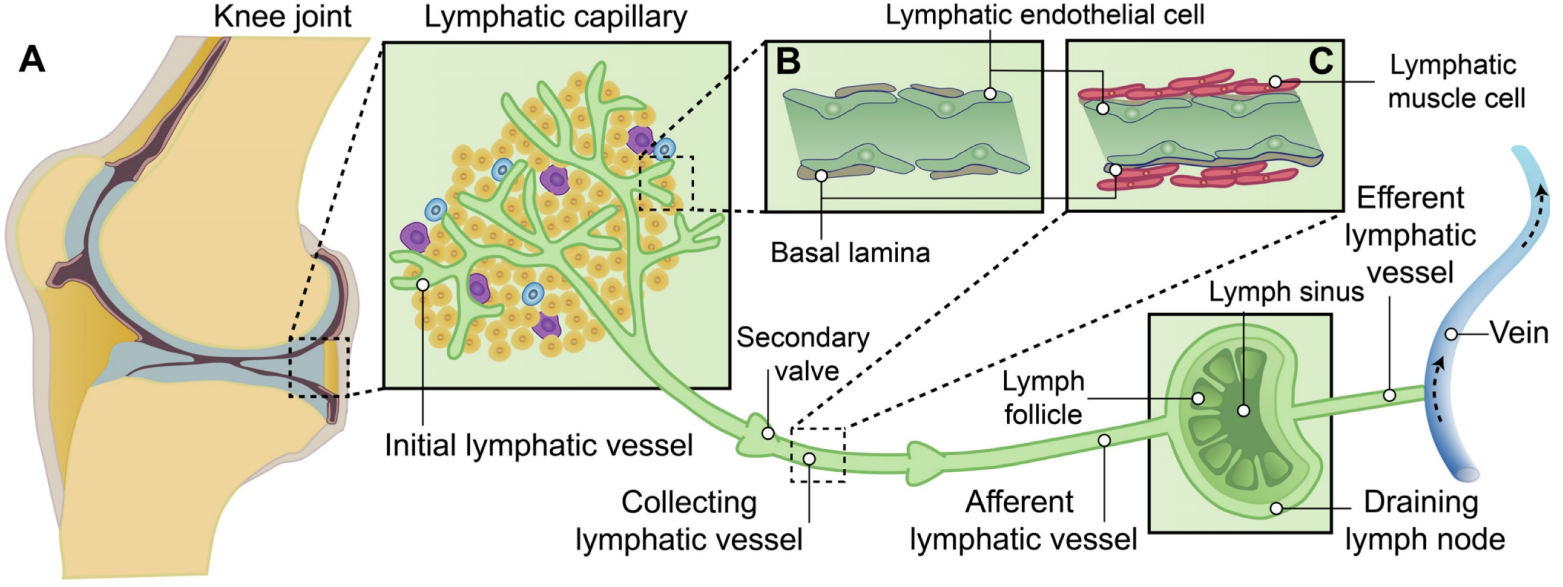
** The hierarchical structure of synovial lymphatic system. (A)** Synovial lymphatic system in joints start from the lymphatic capillary, also named as initial lymphatic vessels. Collecting lymphatics with anti-flowback valves (secondary valve) converge on draining lymph nodes, and then drain lymph to the venous system. **(B)** Initial lymphatic vessels are composed of a single layer of lymphatic endothelial cells and enveloped with discontinuous basal lamina. **(C)** Collecting lymphatic vessels are composed of a continuous basement membrane, a single layer of lymphatic endothelial cells and one or more layers of continuous lymphatic muscle cells.

The lymphatic system is located in close proximity to the venous network, which plays crucial roles in conducting surveillance, and facilitating lipid absorption 13-15. In brief, lymphatic vessels originate from preexisting blood vessels, which initiate from the lymphatic capillary, also known as initial lymphatic vessels, in the peripheral regions of the body. Subsequently, they converge through collecting lymphatics towards draining lymph nodes (DLNs), ultimately facilitating lymph drainage into the venous system via the right lymphatic trunk and thoracic duct 14, 16, 17 (Figure 1).

**Figure 2:**
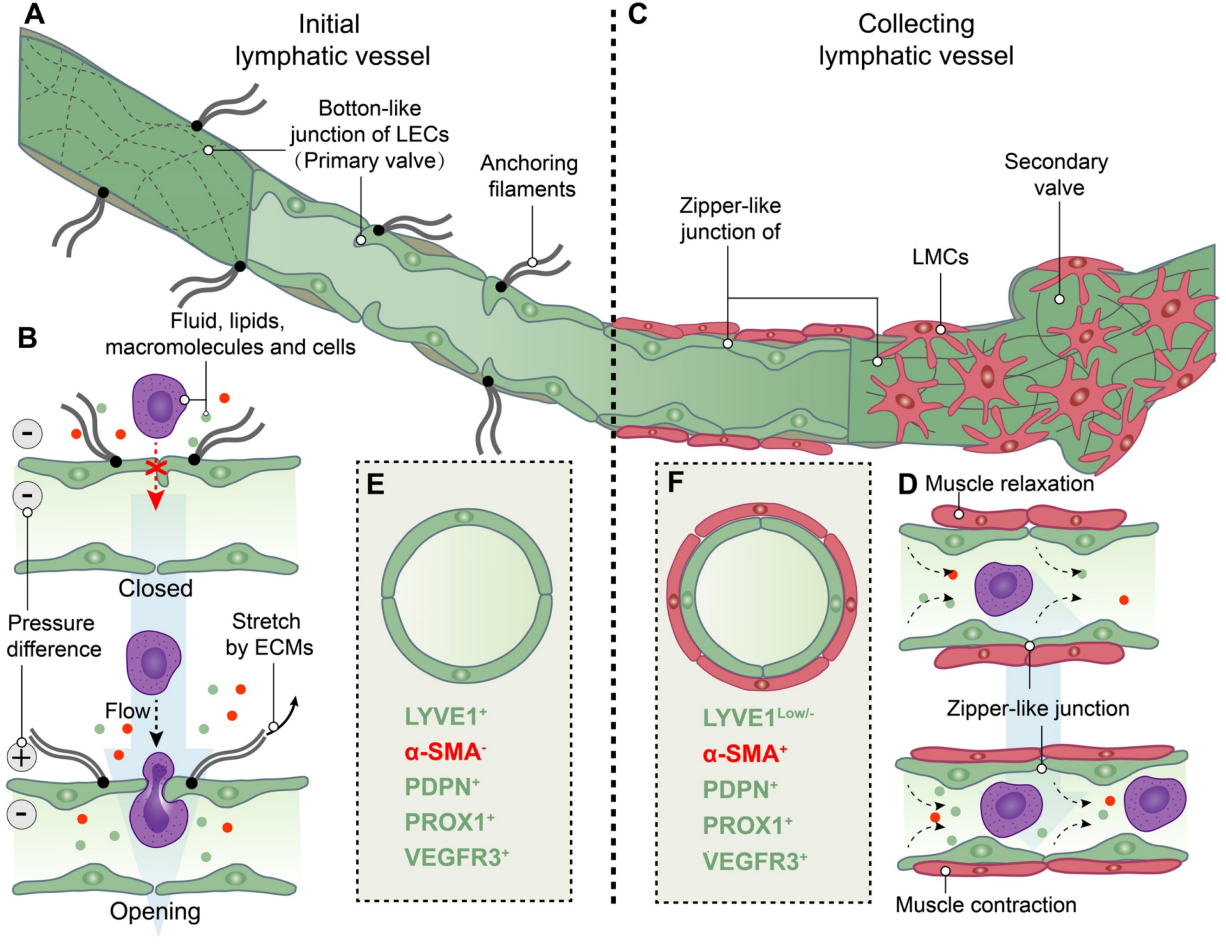
** Structure and function of initial and collecting lymphatic vessels. (A)** Lymphatic endothelial cells **(LECs)** of initial lymphatic vessels directly connected to the surrounding extracellular matrixes **(ECMs)** by anchoring filaments, and LECs are connected by overlapped button-like junctions that act as primary valves. **(B)** When the internal and external pressure difference of vessels or/and the stretch force by ECMs on LECs are existed, primary valves not only permit initial lymphatic vessels to become highly permeable to fluid, lipids, macromolecules and even cells, but also prevent the backflow of these factors into tissues. **(C)** Collecting lymphatic vessels are composed of LECs tightly connected by zipper-like junctions, and lymphatic muscle cells **(LMCs)**. In addition, secondary valves are specialized to prevent the flowback of lymph.** (D)** LMCs drive the contraction of collecting lymphatic vessels to move lymph forward. **(E)** Initial lymphatic vessels are composed of LECs with positive expression of lymphatic vessel endothelial hyaluronan receptor 1 **(LYVE1)**, podoplanin **(PDPN)** and prospero homeobox 1 **(PROX1)** and vascular endothelial growth factor receptor 3 (**VEGFR3**), but not α-smooth muscle actin **(α-SMA)**-positive muscle cells. **(F)** Collecting lymphatic vessels have the low levels of LYVE1 expression compared to initial lymphatic vessels and the positive expression of a-SMA, PDPN, PROX1 and VEGFR3.

The formation of lymph takes place within the lymphatic capillaries upon the influx of interstitial fluid into these vessels. The initial lymphatic vessels in the human lymphatic capillary are slender-walled structures measuring approximately 35-70 μm in diameter and cul-de-sac in nature 18. The initial lymphatic vessels consist of a monolayer of lymphatic endothelial cells (LECs) enveloped by an interrupted basal lamina 13, 19, 20. LECs are firmly anchored to the elastic fibers in the surrounding tissue through anchoring filaments, facilitating the stretching and extension of lymphatic capillaries alongside extracellular matrix and elastic fibers under elevated interstitial fluid pressure 13, 19-23. Moreover, LECs possess distinctive "button-like" junctions that facilitate the formation of highly permeable vessels, allowing for the transmigration of fluids, lipids, macromolecules and even cells 24. Additionally, certain adjacent LECs within the lymphatic capillary exhibit overlapping regions and form the primary lymphatic valve, which serves as a crucial mechanism to prevent backflow of lymph. In the context of edema formation, augmented interstitial fluid pressure induces the opening of "button-like" junctions between LECs, thereby promoting enhanced generation and drainage of lymph within the lymphatic capillaries 13, 21-27 (Figure 2A-B).

Lymphatic outflow occurs through lymphatic capillaries, which converge into the collecting lymphatic vessel. The collecting lymphatic vessel is characterized by a continuous basement membrane, LECs connected via zipper-like junctions, and distinct lymphatic muscle cells (LMCs) that differentiate it from the initial lymphatic vessels in lymphatic capillary. Additionally, the collecting lymphatic vessel is equipped with secondary valves, each consisting of two layers of LECs. These secondary valves possess the ability to open or close in response to periodic fluctuations in fluid pressure, effectively preventing lymph backflow 13, 16, 19, 23, 24. The flow of lymph in collecting lymphatic vessels is dependent on both intrinsic and extrinsic pumps. The intrinsic pump relies on the coordinated contraction or relaxation of LMCs, while the extrinsic pump is driven by the cyclical compression and expansion of lymphatics due to surrounding tissue forces 21, 24, 28. The lymphatic pump and the lymphatic valves collaborate to counteract the gravitational force on lymph its smooth propulsion. Consequently, modulation of LMCs function can regulate lymph flow by modifying both pumping force and outflow resistance 13, 24 (Figure 2C-D).

Following the collection by lymphatic vessels, lymph traverses the afferent lymphatics and reaches DLNs. Within the lymphatic sinuses of DLNs, an autoimmune response is initiated 15. Subsequently, lymph exits these nodes via efferent lymphatic vessels to enter into the circulation of lymph.

### 1.2 Assessment and visualization of lymphatic vessel distribution within joints

Analogous to the circulatory system, lymphatic vessels are distributed across mammalian tissues. To assess and visualize lymphatic vessels, imaging and histological approaches are employed in the investigation. Imaging modalities, such as near-infrared indocyanine green (NIR-ICG) imaging, contrast-enhanced magnetic resonance imaging (CE-MRI), and power Doppler ultrasound (PD-US), enable the comprehensive assessment of lymphatic vessel and lymph node morphology and function in joints 29. Further investigation of the distribution of the lymphatic system within the synovial joint necessitates histological observation, as distinguishing between lymphatic and vascular micro-vessels in histological sections at the light microscope level poses significant challenges. The ideal positive or negative marker should be exclusively localized within lymphatic vessels, rather than relying on relative differences in expression levels between blood and lymphatic vessels 30. Furthermore, it is essential to distinguish between initial and collecting lymphatic vessels. Therefore, the visualization of lymphatic vessels relies on immunostaining markers for both LECs and LMCs (Figure 2E-F). Lymphatic vessel endothelial hyaluronan receptor 1 (LYVE1), podoplanin (PDPN), prospero-related homeobox 1 (PROX1), and vascular endothelial growth factor receptor 3 (VEGFR3) are commonly employed for immunostaining of LECs, while α-smooth muscle actin (αSMA) is utilized for labeling of LMCs 31, 32. LYVE1 is a widely used lymphatic marker, particularly of LECs with the absence of LMCs coverage in initial lymphatic vessels 33, 34. The expression of PDPN and PROX1 is specific to LECs of lymphatic vessels rather than blood endothelial cells (BECs), and providing a crucial molecular distinction between LEC progenitors located within the veins and those emerging outside the cardinal veins 35. Additionally, our group and other researchers have utilized the genetic model of LEC-specific inducible mouse, Prox1-Cre^ERT2^ mice 36, to investigate lymphatic vessels 37, 38. During early development, VEGFR3, also named as Fms-like tyrosine kinase 4 (FLT4), is expressed in both BECs and LECs, but becomes restricted to LECs in adults 39. The PROX1-VEGFR3 feedback loop plays a crucial role in the specification and maintenance of LECs 40, 41. Additional markers, such as 5`-nucleotidase, cluster of differentiation (CD)31, CD34 and pathologische anatomie Leiden-endothelium (PAL-E), are selected based on specific research objectives 30. The conjugation of fluorescent dyes with these markers is noteworthy, as they are highly expressed in initial lymphatic vessels or collecting lymphatic vessels 42-46. To obtain more accurate highlights, multiple-immunostaining is employed. For instance, vessels that are PDPN^+^/α-SMA^-^ are classified as initial lymphatic vessels, while those that are PDPN^+^/α-SMA^+^ are considered collecting lymphatic vessels. On the other hand, lacking PDPN but expressing α-SMA (PDPN^-^/α-SMA^+^) are identified as blood vessels 43, 46, 47.

In synovium of human joints, PDPN^+^ CD45^-^ CD31^-^ synovial fibroblasts were identified in RA and OA patients 48. The presence of PDPN^low+^/LYVE1^low+^/PROX1^low+^ BECs has been observed in chronic skin inflammation 49. Furthermore, the expression of LYVE1 and VEGFR3 has been detected in BECs from patients with OA 50 and RA 51, respectively. LYVE1 is also expressed in some synovial tissue macrophage subsets 52, 53. Therefore, the identification of lymphatic vessels in synovium necessitates the utilization of multiple markers, taking into account their tissue localization and morphology, as well as the specific tissue microenvironment and cell types involved. At the cellular tissue level, the synovial membrane in joints consists of two distinct layers: an inner layer and an outer layer positioned beneath it. Lymphatic vessels are predominantly found in the outer layer of the synovium, characterized by a loosely arranged collagenous extracellular matrix 54, while they are absent in the lining layer composed of macrophage-like and fibroblast-like synoviocytes 55. In order to visualize the ensemble of the lymphatic vasculature, whole-slide digital imaging systems were employed for scanning images of stained joint tissue sections and quantifying the number and size of lymphatics, thereby enhancing credibility and comparability 43, 56. Recently, a novel immunolabeling and imaging technique of intact bone tissues was employed on a light-sheet microscopy platform to investigate the lymphatic vessels involved in bone regeneration in mice 31.

Based on the aforementioned advancements in lymphatic vessel detection technology, the elucidation of lymphatic vessel distribution within joints has been consistently unveiled. Lymphatic vessels were identified within the stratified connective tissues surrounding the fetal cartilaginous knee joint tissues through immunostaining with PDPN and LYVE1 antibodies 57. In adult mice, the presence of both lymphatic capillaries and collecting lymphatic vessels, as indicated by positive staining for PDPN and α-SMA, was observed in various soft tissues including capsule, ligaments, fat pads, muscles, and the patellar region; however, they were not detected in cartilage tissues 43, 46. Moreover, lymphatic vessels have been identified within the periosteum of long bones 43. Previous studies have demonstrated the limited efficacy of immunohistochemical staining in identifying vessels within the bone marrow 56, 58, suggesting that fluid clearance predominantly occurs through venous sinusoids rather than lymphatics. The present study provides strong evidence for the presence lymphatic vessels in mouse and human bones, which are involved in supporting bone regeneration following injury 31. Therefore, lymphatic vessels are extensively distributed throughout the various tissues of the joint, excluding articular cartilage. The employment of additional high-resolution techniques is essential for subsequent research, which aims to evaluate the distribution of lymphatic vessels within the joint and discern diverse subpopulations.

### 2.1 Lymphatic vessels and RA

RA is a chronic autoimmune disorder that primarily affects the joints, leading to persistent inflammation and progressive damage. This results in the release of inflammatory mediators and the activation of immune cells, which further exacerbate the inflammation. Inflamed joints in RA patients are typically characterized by a marked increase in the number of activated and infiltrated immune cells, such as macrophages, lymphocytes, and plasma cells. These cells play a crucial role in the progression of joint inflammation, as they are responsible for producing and releasing various mediators, including cytokines, chemokines, and enzymes 59, 60. Some of the most important cytokines involved in RA pathogenesis and progression are tumor necrosis factor (TNF)-α, interleukin (IL)-1, and IL-6. These inflammatory mediators can induce synovial inflammation and vasodilation, leading to joint pain, swelling, and functional impairment 61. Both clinical studies and animal models suggest that lymphatic vessels likely play a crucial role in the clearance of these inflammatory cells and mediators from the inflamed synovium.

### 2.2 Animal models of RA in the investigation of lymphatic vessels

In classical animal models of RA, the utilization of K/BxN mice 45, 62, collagen-induced mice 63 and TNF-transgenic (TNF-Tg) 62, 64-69 mice was established to investigate the role of lymphatic vessels within joints. In K/BxN mice expressing both the T cell receptor (TCR) transgene KRN and the MHC class II molecule A, leading to recognition of glucose-6-phosphate-isomerase as an antigen by both T cells and B cells, a joint-specific autoimmune phenotype with numerous characteristics reminiscent of RA is observed 70. Collagen, a critical autoantigen observed in human RA, is utilized to generate collagen-induced mice through active immunization via intradermal injection with heterologous type II collagen (CII) 71. These two animal models exhibit a propensity to replicate the abrupt onset of RA. TNF-α plays a pivotal role in the pro-inflammatory cytokine cascade, and its activation elicits systemic inflammatory responses in RA 72. The TNF-Tg mice, characterized by the overexpression of human TNF-α, exhibit a range of features reminiscent of those observed in patients with RA, including impaired mobility, joint inflammation, synovial hyperplasia, cartilage degradation, and osteoporosis; however, they do not manifest any discernible developmental abnormalities 73.

### 2.3 Vascular endothelial growth factor C (VEGF-C)/vascular endothelial growth factor receptor 3 (VEGFR3) signaling pathway in lymphangiogenesis of RA

**Figure 3:**
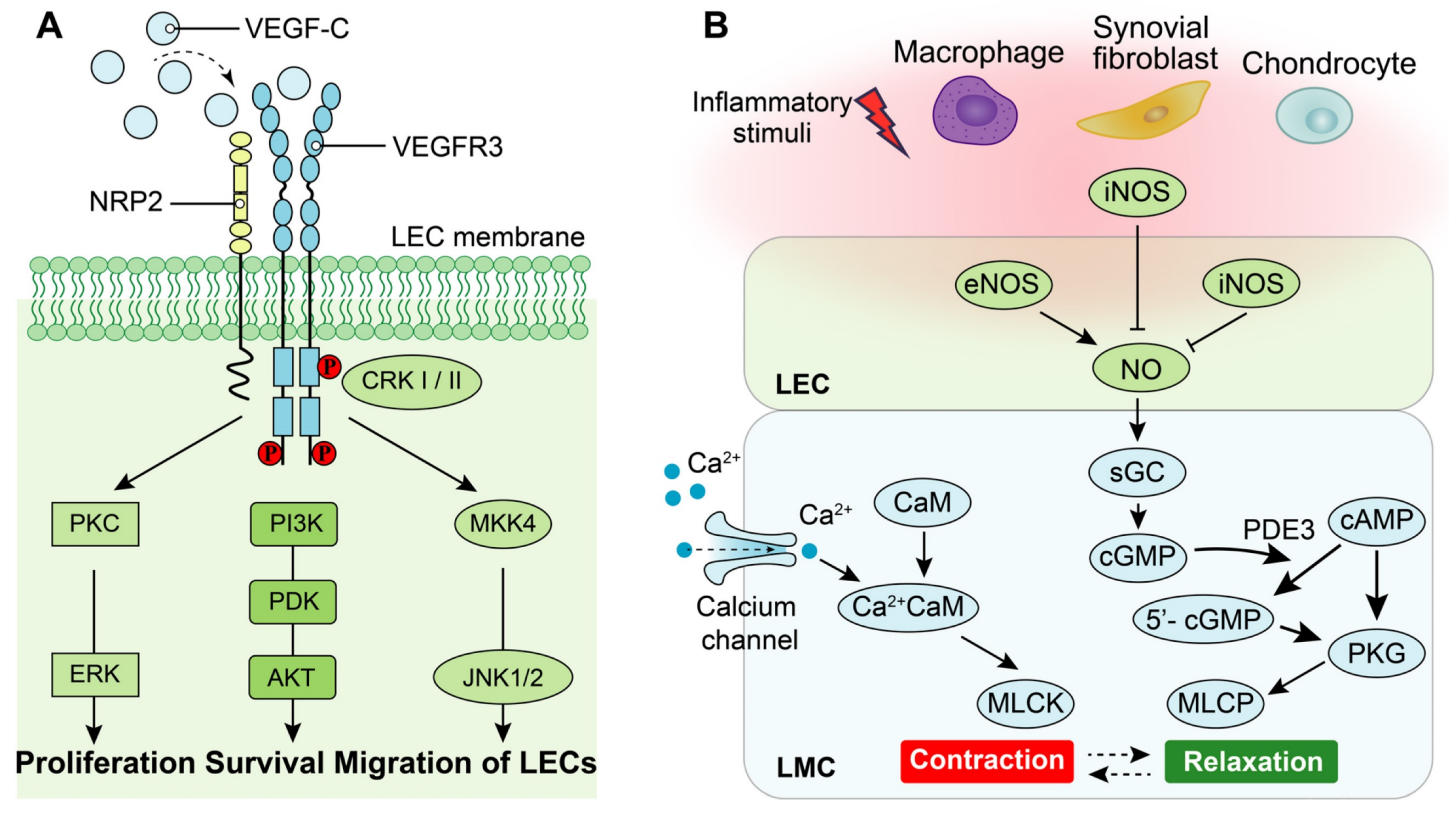
** The role of VEGF-C/VEGFR3 and nitric oxide synthase (NOS) signaling pathways on LECs and LMCs. (A)** Neuropilin-2 **(NRP2)** promotes activation of VEGFR3 in LECs in response to VEGF-C. Phosphorylation of VEGFR3 activates the downstream PKC/ERK, PI3K/AKT, and JNK signaling pathways to regulate proliferation, survival and migration of LECs. **(B)** Under normal condition, calcium signaling induces LMC contraction, whereas LMC relaxation is regulated by NOS signaling. Endothelial NOS **(eNOS)** of adjacent LECs produces NO to inhibit LMC contraction which allows relaxation of lymphatic vessels. Under inflammatory condition, inducible NOS **(iNOS)** in adjacent LECs, activated-adherent macrophages and/or synovial fibroblasts and chondrocytes of joints suppresses NO function to disorder the cycle of lymphatic vessel contractions. **PKC:** Protein kinase C,** ERK:** Extracellular regulated kinases, **PI3K:** Phosphoinositide 3-kinase, **PDK:** Phosphoinositide 3-kinase, **AKT:** Protein kinase B,** CRK-I/II:** CT10 regulator of kinase adaptor proteins I/II, **MKK4:** Mitogen-activated protein kinase 4, **JNK:** c-Jun N-terminal kinase, **sGC:** Soluble guanylate cyclase, **cGMP:** Cyclic guanosine monophosphate,** cAMP:** Cyclic adenylic acid, **PKG:** Protein kinase G, **MLCK:** Myosin light chain kinase,** MLCP:** Myosin light chain phosphatase,** CaM:** Calmodulin.

Lymphangiogenesis is a complex process regulated by cytokines and other factors, particularly the VEGF family 20, 74. The VEGF family comprises essential regulators in the angiogenic process, including VEGF (also known as VEGF-A with multiple functional isoforms), VEGF-B, VEGF-C, VEGF-D, VEGF-E, VEGF-F, and placenta growth factor (PIGF) 75. These ligands of the VEGF family activate signaling pathways by binding to tyrosine kinase receptors called vascular endothelial growth factor receptors (VEGFRs), which consist of three subtypes: VEGFR1, VEGFR2 and VEGFR3^76^. While both VEGFR1 and VEGFR2 are primarily associated with the regulation of angiogenesis, the signaling pathway of VEGFR3 plays a central role in the regulation of lymphangiogenesis 76. Specifically, high-affinity binding between the ligands of VEGF-C/VEGF-D and their receptor VEGFR3 induces receptor dimerization and phosphorylation, which triggers the downstream signaling pathways that promote lymphangiogenesis 77. Additionally, interaction between neuropilin (NRP)2 and VEGFR3 could improve lymphangiogenesis via mediating proper lymphatic vessel sprouting upon VEGF-C stimuli 77, 78. The downstream signaling pathways activated by VEGF-C/VEGFR3 include mitogen-activated protein kinase/extracellular signal-related kinase (MAPK/ERK), phosphatidylinositol 3-kinase/protein kinase B (PI3k/AKT), as well as Jun N-terminal kinase1/2 (JNK1/2) pathways 79, 80. Activation of these downstream signaling pathways leads to proliferation, survival, and migration of LECs along with remodeling of lymphatic vessels (Figure 3A).

Previous studies have demonstrated that VEGF-C/VEGFRs plays an important role in RA. Firstly, VEGF-C and its receptors VEGFR3 and 2 exhibit high expression levels in arthritic synovial tissue compared to healthy controls, serving as major regulators in lymphangiogenesis. Additionally, immunohistochemical staining reveals the presence of positively stained cells for VEGF-C protein primarily located within synovial lining cells such as synovial endothelial cells and fibroblasts 51, 81. Macrophages exhibit abundant expression of VEGF-C and VEGFR3 in an inflammatory environment of RA^82^. Furthermore, a significant elevation of VEGF-C levels was observed in synovial fluid from patients with RA, exhibiting a strong positive correlation with TNF-α levels 83. Potential molecular mechanisms were also researched in this field. In inflammatory microenvironment, PROX1 is activated by nuclear factor-κB (NF-κB) pathway and then regulate the VEGFR3 promoter activity, which strength the expression of receptors in LECs and the susceptibility of VEGF-C 84. Moreover, VEGF-C/VEGFR3 signal pathway also suppress Toll-like receptors 4 (TLR4)/NF-κB pathway 85.

### 2.4 Nitric oxide synthase (NOS) signaling pathway in LMCs and the contraction of lymphatic vessels of RA

LMCs in lymphatic vessels exhibit a unique composition of both smooth and striated muscles, endowing them with the ability to generate robust rhythmic contractions 13 24. The contractile force of lymphatic vessels is primarily generated by the contraction of LMCs, which is tightly regulated by the interplay between cellular calcium dynamics and contractile proteins 24, 86 (Figure 3B). Specifically, stretch-induced contractions are mediated through the activation of L-type and T-type Ca^2+^ channels. Upon pacemaker-generated action potentials in lymphatic muscle cells, voltage-dependent Ca^2+^ channels open, allowing extracellular Ca^2+^ influx into LMCs. Subsequently, binding of Ca^2+^ to calmodulin triggers muscle contraction 86. Moreover, the relaxation of LMCs is predominantly regulated by the nitric oxide (NO). In the lymphatic system of joints, the synthesis of NO is predominantly orchestrated by LECs and originates from endothelial NO synthase (eNOS) of LECs, while inducible NO synthase (iNOS) generated either directly or indirectly by LECs 65, 68, macrophages 65, 87, chondrocytes 88, 89 and fibroblasts 87. NO activates cytoplasmic guanylate cyclase within LMCs to reduce vessel tone via cyclic guanosine monophosphate (cGMP)-dependent mechanisms 90. Furthermore, NO inhibits intracellular Ca^2+^ entry from internal stores 91.

The expression of iNOS is primarily induced by inflammation, leading to the abundant production of NO, which plays a crucial role in the pathogenesis of inflammatory arthritis 92. Previous study demonstrated that the iNOS mRNA level in LECs of efferent lymphatic vessels from TNF-Tg mice was significantly elevated by 8-fold compared to control mice 66. The systolic function in ex vivo of popliteal lymphatic vessels from TNF-Tg mice is significantly impaired, which is NO synthase dependent 64. Additionally, TNF directly induces LMC apoptosis through the production of NO by LECs 67. The activation of NF-κB/iNOS signaling pathway in LECs by TNF leads to impaired lymphatic contraction function and subsequent production of NO mediated by iNOS 68. In addition to LECs, macrophages stimulated by lipopolysaccharide significantly upregulate the expression of iNOS and induce lymphatic vessel dilation 93. The expression of iNOS is also significantly upregulated in chondrocytes during inflammatory arthritis; Moreover, excessive production of NO can exacerbate inflammation by impeding matrix synthesis and facilitating its degradation 88. Therefore, inflammation induces the upregulation of iNOS and leads to excessive production of NO, which impairs LMCs contraction and diminishes the function of lymphatic drainage.

### 2.5 Changes of lymphatic vessels during RA processes

Clinical studies have demonstrated a significant augmentation of lymphatic vessels within the arthritic synovial membrane in comparison to healthy controls 94, 95. The present finding is further substantiated by the observation of significantly increased numbers and sizes of lymphatic vessels in synovial tissues from TNF-Tg mice model and K/BxN mice model 96. Moreover, the whole-slide imaging system demonstrated an augmentation in capillary lymphatics and a reduction in collecting lymphatics were observed in TNF-Tg mice 46 as well as a mouse model of RA-associated periodontitis 97. In a recent study, it was further observed that dilated capillary lymphatic vessels exhibited a decreased number of branch points compared to the wild control group. Additionally, collecting lymphatic vessels demonstrated decreased coverage of LMCs and impaired drainage function. Furthermore, elevated levels degenerative and apoptotic LMCs were observed 67.

**Figure 4:**
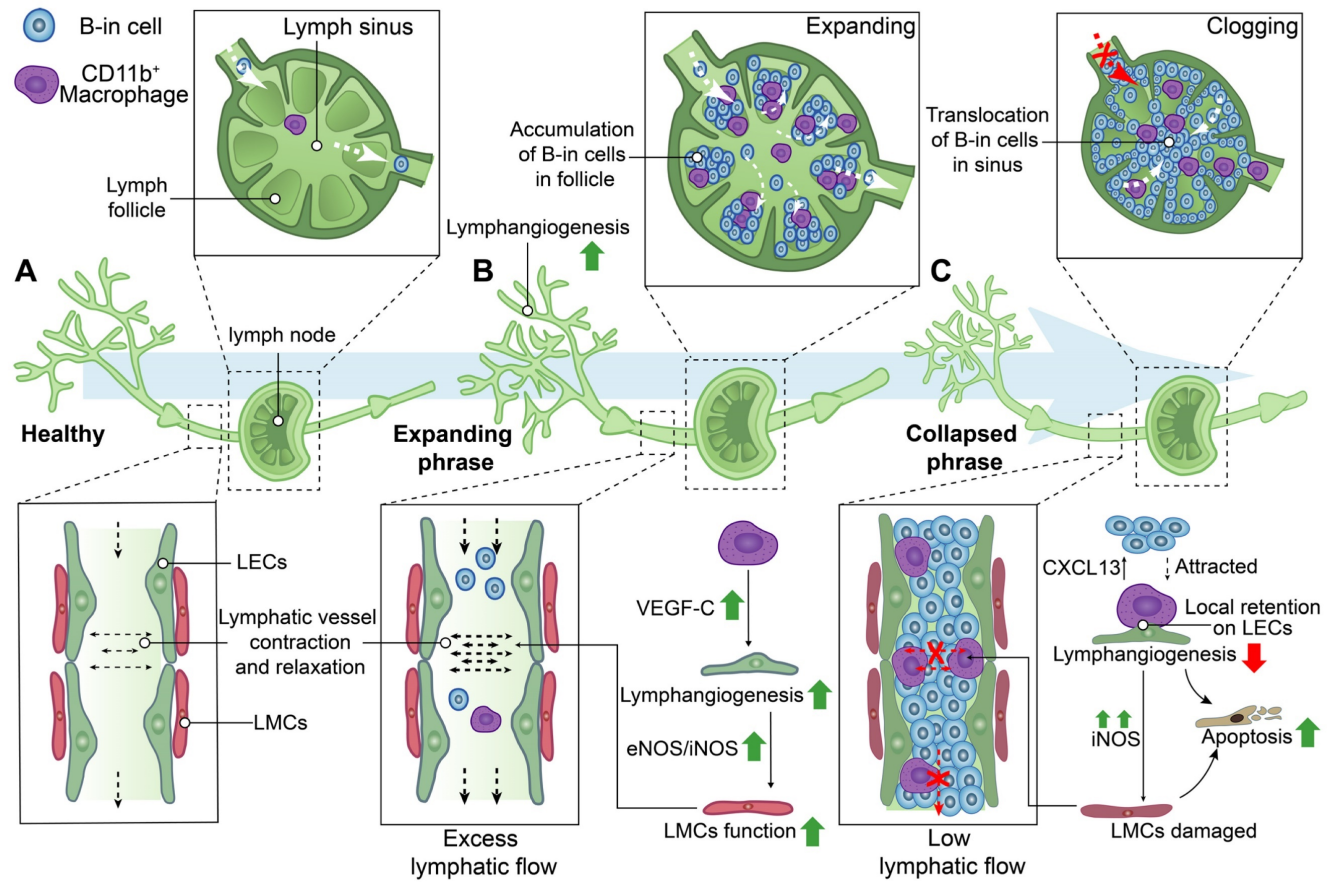
** Schematic diagram demonstrating lymphatic phenotypes in the development of rheumatoid arthritis.** Two different phenotypes of the lymph node, called expanding and collapsed phrase, were observed during rheumatoid arthritis development. **(A)** Lymphatic capillaries retrieve lymph and inflammatory factors which flow into lymphatic collecting vessels and be transported to lymph nodes by the flow force of lymphatic collecting vessels contraction. **(B)** During expanding phrase, lymphangiogenesis in the lymphatic system facilitates removal of inflammatory factors and lymph to the expanding lymph nodes with the accumulation of B-in cells in follicles. Macrophages are involved in regulating lymphangiogenesis and the contraction of lymphatic muscle cells **(LMCs)**. **(C)** During collapsed phrase, lymph nodes have collapsed in volume owing to damaged LMC with low lymphatic flow, the disorders of lymphangiogenesis, and the cloggy of B-in cells in sinus. Local retention of CD11b^+^ macrophages on lymphatic endothelial cells **(LECs)** are involved in regulating the attraction of B-in cells, lymphangiogenesis and the function of LMCs. **VEGF-C**: Vascular endothelial growth factor C, **eNOS:** Endothelial nitric oxide synthase, **iNOS:** Inducible nitric oxide synthase, **CXCL13:** Chemokine C-X-C motif ligand 13.

In addition to the modifications in the distribution and structure of lymphatic vessels, alterations in the clearance function of lymphatic vessels were also observed in the processes of RA (Figure 4). NIR-ICG lymphatic imaging revealed a significant increase in the frequency of lymphatic vessel contractions with the enlargement of DLNs in K/BxN mice during the acute phase of RA, subsequently normalizing the collapse of expanding DLNs during the chronic phase 45. Therefore, the progression of RA is characterized by the presence of two distinct phenotypes of DLNs, namely expanding and collapsed DLNs.

During the expanding phase of DLNs, the augmented DLN volume is associated with an upregulation of lymphangiogenesis, infiltration of CD11b+ macrophages, and accumulation of a distinct subset of B cells characterized by high expression levels of CD23, CD21, IgM, IgD, and CD1d in inflamed nodes, also named B-in cells 45, 62, 96, 98-101. CD11b^+^ macrophages actively contribute to lymphangiogenesis, play a crucial role in maintaining lymphatic function, and exhibit the potential to differentiate into osteoclasts as osteoclast precursors (OCPs) during inflammatory conditions 96, 102, 103 . CD11b^+^ macrophages alone possess the capability to generate lymphatic endothelial marker-expressing tube-like structures, including LYVE-1 and PDPN, in inflamed stromata of murine corneas 104. Additionally, CD11b^+^/Gr-1^+^ macrophages, which exhibit a high expression of VEGF-C, were found to extensively infiltrate the inflamed skin and DLNs in a bacterial pathogen-induced acute inflammation model 105. Furthermore, CD11b^+^ macrophages represent the primary source of VEGF-C in TNF-Tg mice, and neutralization of VEGFR3 resulted in a reduction in the number of CD11b^+^ cells expressing VEGF-C in DLNs 99. These findings suggest that CD11b^+^ macrophages play a crucial role in promoting lymphangiogenesis, particularly during the expanding phase of DLNs.

During the stage of collapsed DLNs, a rapid decrease in volumes of DLNs was observed, which is associated with extensive bone loss in adjacent knee joints 45, 62, 64, 65, 99,97. Lymphatic sinus serves as an essential conduit for lymph drainage 13. B-in cells reside in the peripheral follicular regions during the expanding phase of DLNs, subsequently relocating to the lymphatic sinus during the collapsing phase. This translocation of B-in cells obstructs both lymph channels and the cavity of the lymphatic sinus, resulting in DLNs collapse and significantly diminished lymphatic drainage from inflamed joints. These findings elucidate the concurrent synovitis erosions, accumulation of B-in cells, and asymmetric onset of arthritis observed in TNF-Tg, K/BxN mice and RA patients 62, 98, 100, 101. Noteworthy, the lymphatic vessels afferent to expanding DLNs exhibited a high-velocity flow of CD11b^+^ macrophages, whereas the lymphatic vessels afferent to collapsing DLNs showed stationary CD11b^+^ macrophages due to the loss of intrinsic lymphatic contractions and passive flow 106. The local retention of CD11b^+^ macrophages lead to the damage of LECs and LMCs 82 93. Additionally, a gradient of chemokine C-X-C motif ligand 13 (CXCL13), released by static, aggregated, and activated macrophages in DLNs, attracts the migration of B-in cells 107. CD11b^+^ macrophages also serve as a significant source of osteoclasts and are thus referred to as OCPs 96. A study has reported a 4-7-fold increase in OCPs in the peripheral blood and spleen of TNF-Tg mice, which can be reversed by anti-TNF therapy 102. Thus, on one hand, the increased and localized retention of CD11b^+^ macrophages could attract the migration of B-cells to lymphatic sinus, resulting in the obstruction of lymphatic vessels. On the other hand, these macrophages are directly involved in the extensive bone loss in adjacent knee joints during the collapsed phase of DLNs 108.

### 3.1 Lymphatic vessels and OA

**Figure 5:**
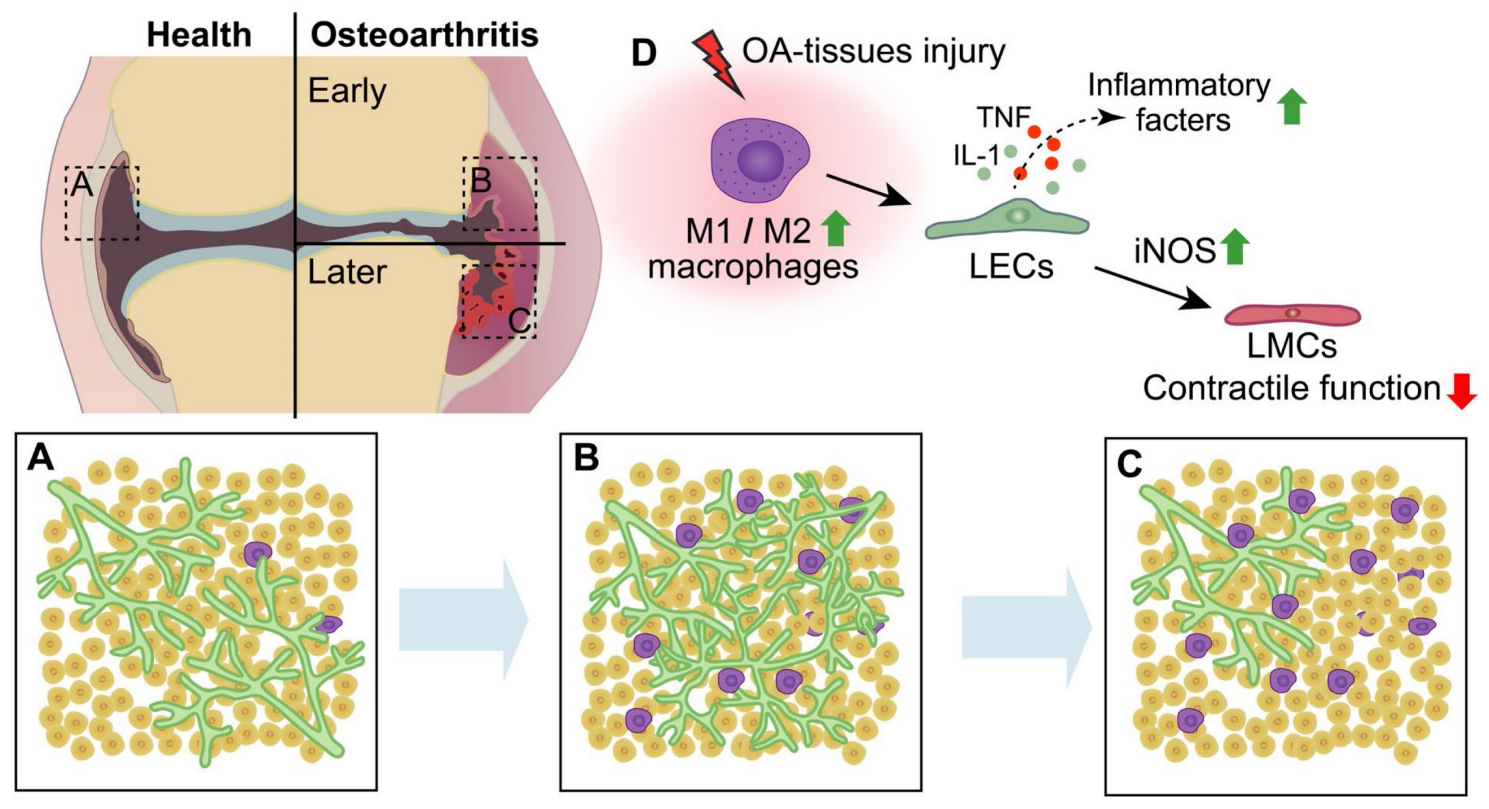
** Schematic diagram demonstrating lymphatic phenotypes in the development of osteoarthritis. (A-C)** In the process of osteoarthritis **(OA)** development, lymphangiogenesis of lymphatic capillaries are observed in the early stage of OA, whereas decreased lymphatic capillaries and impaired lymphatic pumping function are observed in the late stage of OA.** (D)** After OA-tissues injury, M1 macrophages is significantly increased compared to M2 macrophages, which induces the production of lymphatic endothelial cells **(LECs)** inflammatory factors and damages the function of lymphatic muscle cells **(LMCs)** via increasing inducible nitric oxide synthase** (iNOS)** of LECs.

OA is a prevalent joint disorder characterized by the degeneration and damage of the articular cartilage, accompanied by inflammation of the surrounding tissues 109, 110. While patients with OA and RA may experience similar symptoms of joint stiffness and pain, these two types of arthritis differ significantly in terms of their underlying causes. OA is primarily attributed to the gradual mechanical degradation of cartilage and the disorder of subchondral bone due to aging processes, whereas RA is characterized by a disease of autoimmune response. The pivotal role of synovial cells in OA is widely acknowledged, as they actively release a range of inflammatory mediators 109, 111. These inflammatory mediators, acting as pivotal factors, stimulate the synthesis of inflammatory cytokines and matrix degrading enzymes such as matrix metalloproteinases (MMPs) 112 and the a disintegrin and metalloproteinase with thrombospondin motif (ADAMTS) family 113 of proteins in chondrocytes, thereby instigating cartilage destruction and degradation during OA progresses 114. Therefore, the role of inflammation in the pathogenesis of OA has gained increasing consensus among researchers. Similar to RA, the peri-articular lymphatic system plays a significant role in the pathogenesis of OA 12. Although extensive research has been conducted on the involvement of lymphatic vessels in human and murine RA, limited attention has been given to investigating their role in OA (Figure 5).

### 3.2 Dynamic alterations of lymphatic vessels involves in various stages of OA progression

Based on clinical reports, immunohistochemical analysis of synovial specimens from patients with OA revealed an increased presence of lymphatic vessels infiltrated by numerous inflammatory cells across all zones of the synovial membrane 94. These findings imply the potential involvement of synovial lymphatic vessels in the inflammatory cascade underlying OA pathogenesis. On the other hand, dysfunction of microcirculation and lymphatic drainage was observed in samples of OA patients by transmission electron microscopy 115. Furthermore, a study has reported that lymphatic vessel density and the fractional area of LECs in knee synovium sections from patients with OA are lower compared to normal control knees in post-mortem analysis. Additionally, there is a negative correlation between lymphatic vessel density and synovial effusion, which is a characteristic feature of advanced stages of OA development 50. The reduced lymphatic vessel density may contribute to the retention of synovial fluid in OA patients, potentially exacerbating joint inflammation by impairing lymphatic pumping function 116. In addition to the number of lymphatic vessels, the relationship between the severity of OA and the number, size, and central fatty changes of DLNs observed in MR images of patients does not exhibit a linear trend 117. Therefore, these findings suggest that dynamic alterations in the structure and functions of the lymphatic system may play a significant role in various stages of OA progression. Further investigation is warranted to elucidate the long-term mechanisms by which the lymphatic system maintains joint homeostasis during OA progresses.

### 3.3 Synovial lymphatic drainage is impaired during OA progression

In the mice model of meniscal-ligamentous injury (MLI)-induced OA, increased capillary lymphatics and decreased collecting lymphatic vessels in OA joints were detected at 3 months post-MLI by the whole-slide imaging system 46. Further study found significantly increased lymphatic vessels, especially lymphatic capillaries, in the thicker synovial membrane of mice at 12 weeks post-MLI and cartilage-specific knockout of transforming growth factor beta (TGF beta) type II receptor mice at 6 weeks after tamoxifen induction 43. Potentially, an increase in lymphatic capillaries could enhance lymphatic drainage; however, a decline in lymphatic drainage function was also detected in these mice 43, 118. Recent findings suggest that the increased lymphangiogenesis of lymphatic capillaries does not exhibit a positive correlation with lymphatic vessel contractility and transport properties in visceral adipose tissues of rodents and humans with obesity and/or diabetes 119. One potential explanation for this phenomenon is that the emergence of extensively branched lymphatic capillaries in their juvenile stage could elicit a leaky phenotype, thereby hindering their drainage function. The lymphatic capillaries and collecting lymphatic vessels were significantly reduced, leading to impaired lymphatic pumping function; Consequently, lymph clearance was significantly diminished and pro-inflammatory factors accumulated in the knees of both 20-week MLI-induced OA 43 and aging-related OA 120 mice, which is further supported by OA samples from patients 43. Therefore, the above results show that synovial lymphatic drainage is impaired during OA progression.

### 3.4 The collaboration of macrophages and lymphatic vessels regulates OA

Although the predominance of T-lymphocytes and monocyte infiltration, as well as synovitis and concentrations of inflammation mediators in joints, appears to be less pronounced in OA compared to RA, it is widely acknowledged that macrophages play a significant role in the process of joint inflammation and bone destruction in OA 121-124, potentially influencing this process through collaboration with lymphatic vessels. Macrophages are highly adaptable cells that can be categorized into two distinct phenotypes, namely classically activated M1 (pro-inflammatory) macrophages and alternatively activated M2 (anti-inflammatory) macrophages, which exhibit differential responses to microenvironmental stimuli 125. The M1 macrophages secrete substantial quantities of proinflammatory cytokines and mediators, such as TNF-α, IL-1, and IL-6^126^. The M2 macrophages, also referred to as healing macrophages, exhibit an anti-inflammatory phenotype and contribute significantly to tissue repair, remodeling and pro-tumorigenic functions 127, 128. Additionally, the production of VEGF-C by M2 macrophages is associated with lymphangiogenesis in human tumors 129. Macrophages accumulate and undergo polarization (M1 or M2) within the synovium and articular cavity during the progression of OA 130, 131. In the early stage of OA (5-6 weeks post-MLI), synovitis and accumulation of M1 macrophages adjacent to lymphatic vessels were observed in the synovium of mice joints. Furthermore, M1 macrophages play a crucial role in promoting destructive processes by regulating the expression of inflammatory mediators, such as TNF, IL-1 and iNOS, in LECs and synovial lymphatic drainage in inflamed synovial tissue 118. Itch serves as a crucial negative regulator of NF-κB, a pivotal inflammatory signaling pathway, and has been observed to suppress the pro-inflammatory polarization of macrophages and the release of IL-1α, an inflammatory cytokine 132, 133. Global knockout of itch in mice results in severe phenotypes associated with OA and impairs synovial lymphatic drainage through M1 macrophage-induced inflammatory response in LECs 134. In our group, we observed decreased fibroblast growth factor receptor 3 (FGFR3) expression in monocytes derived from patients with OA. Conditional knockout of FGFR3 in macrophages using lysozyme-Cre mice exacerbated joint destruction by promoting synovitis and enhancing macrophage accumulation through CXCL12/CXCR7-dependent chemotaxis in both aging and MLI-induced OA models 135. Given that the secretion of CXCL12 from LECs is crucial for tissue regeneration after injury 31, it is imperative to further investigate the potential interplay between synovial tissue-resident macrophages and lymphatic vessels in OA.

## 4. Potential impact of inflammatory environment on lymphatic vessels in RA and OA

The inflammatory response observed in RA exhibits distinct characteristics compared to that seen in OA 136. RA exhibit a chronic and high-grade systemic inflammation 137, whereas the pathogenesis of OA is characterized by mild inflammation within the synovium 138. Specially, the expression of TNF-α and IL-1β is higher in both the number of cells and levels in the synovium of RA compared to OA, while the OA synovium shows an increased presence of cells expressing the anti-inflammatory marker IL-10 139, indicating a more inflammatory environment in RA compared to OA. Therefore, variations in the inflammatory environment within the synovium of RA compared to OA may exert an influence on the structure and functionality of lymphatic vessels, thereby contributing to disparities in disease pathology and progression.

In the synovium, RA exhibits a higher presence of infiltrating inflammatory cells compared to OA 140. Recently, the application of high-resolution techniques has provided a comprehensive understanding of the cellular composition within the synovium in both RA and OA 48. In this study, OA serves as a reference for controlling the highly inflammatory RA synovium, establishing the maximum levels of OA inflammation as the threshold for defining leukocyte-rich or leukocyte-poor RA. Leukocyte-poor RA synovium exhibits a closer resemblance to OA synovium in terms of the proportion of leukocytes and PDPN^+^ cells, including lymphatic vessels and fibroblasts within the tissue, suggests leukocyte-poor RA and OA have similar inflammatory states to some extent. In contrast, leukocyte-rich RA synovial tissue exhibits a profusion of monocyte, B and T cell infiltrates alongside comparatively fewer PDPN^+^ cells, suggesting a pronounced impairment in lymphatic vessel drainage function during RA progression that requires further investigation.

Various subgroups of macrophages significantly contribute to the inflammatory responses in both RA and OA. Studies have shown that macrophages are directly involved in the regulation of lymphangiogenesis and lymphatic vessel function in a variety of diseases 141, 142. In the synovium, the co-localization of M1 macrophages and PDPN^+^ LECs in OA, along with elevated gene expression levels of pro-inflammatory factors in LECs 118, suggests the influence of macrophages on LEC function. Furthermore, transcriptional changes of single-cell RNA-sequencing on the joints of mice with RA progression exhibited an elevation in inflammatory monocytes, accompanied by a decrease in LMCs of lymphatic vessels and M2 macrophage populations 143. Of note, the synovium of leukocyte-rich RA exhibits an increased population of monocytes polarized towards IL-1β^+^ and interferon (IFN)-γ-activated secreted phosphoprotein (SPP1)^+^ pro-inflammatory M1-like macrophages compared to OA synovium, where the majority of macrophages are nuclear protein 1 (NUPR1)^+^ and Mer proto-oncogene tyrosine kinase (MerKT)^+^ M2-like polarized in response to the unique homeostatic requirements of the synovium 48, 52. Thus, further investigation into the interactions between different macrophage subgroups in RA and OA and lymphatic vessels is necessary to elucidate their involvement in the distinct states of the inflammatory process.

## 5. Targeting the lymphatic vessels as a potential therapeutic strategy

The statement highlights the potential for distinct regulatory mechanisms and varying treatment approaches in addressing disorders of lymphatic vessels associated with RA and OA. In accordance with the pathogenesis of RA, potential lymphatic-modulating treatments for RA development include strategies to anti-inflammation and restore lymphatic vessel contraction, as well as remove inflammatory cells to unclog lymphatic vessels. Additionally, it is evident that reduced lymphatic vessel numbers and impaired lymphatic contraction contribute to compromised lymphatic drainage, which correlates positively with the severity of arthritic inflammation in both RA and OA. Therefore, enhancing lymphangiogenesis and improving lymphatic draining function represent potential viable treatment strategies (Table 1).

**Anti-TNF treatment.** As previously mentioned, TNF-α plays a significant role in the pathogenesis of RA 69. TNF-Tg mice, which overexpress human TNF-α, exhibit numerous features observed in RA patients. Consequently, therapeutic interventions targeting TNF-α blockade have been extensively investigated and implemented in clinical practice 144-147. The systemic administration of anti-TNF therapy leads to a reduction in macrophage numbers through the promotion of lymphatic vessel recovery and enhanced lymphatic contraction, ultimately resulting in improved lymphatic drainage of a RA mouse model and provides the most significant pain relief for symptomatic knee joints in RA patients 82, 98, 101, 148. Furthermore, the notable reduction of inflammatory responses in the synovium through anti-TNF treatment is not attributed to an increase in macrophage apoptosis or impaired monocyte influxion 149-151. Although systemic anti-TNF therapy is generally considered efficacious in the treatment of RA, a significant proportion of patients (about 40%) exhibit inadequate response 152, 153. Considering the crucial role of lymphatic drainage in RA, localized delivery of anti-TNF drug targeting the immune system through reversing lymphatic dysfunction and reducing RA-associated swelling may exhibit more favorable effects compared to systemic administration in a collagen-induced RA 154. For OA treatment, the efficacy of TNF inhibition strategy was evaluated in two clinical trials, wherein Adalimumab and Etanercept (TNF inhibitors) were administered for 12 weeks and 24 weeks, respectively. However, no significant differences in pain control were observed in patients with hand OA 155, 156.

**Bortezomib treatment.** The proteasome inhibitor Bortezomib has been approved by the US Food and Drug Administration for the first-line treatment of patients diagnosed with multiple myeloma 157. Studies conducted on murine models of experimentally induced arthritis have demonstrated the efficacy of proteasome inhibitors, such as MG132 158 and Bortezomib 159, 160, in ameliorating inflammatory joint manifestations through modulation of the NF-κB signaling pathway. In MLI-induced OA mouse model, intra-articular administration of Bortezomib significantly improves synovial lymphatic drainage, decreases numbers of M1 macrophages and the inflammatory gene expression by LECs 118. Thus, Proteasome inhibitor, Bortezomib, may potentially serve as a novel therapeutic approach for restoring synovial lymphatic function in arthritis. However, further investigation through human clinical trials is necessary to validate its therapeutic potential.

**B cell depletion therapy.** The translocated B-in cells from the DLN follicles into the sinuses during the stage of collapsed DLNs are believed to exert a mechanical hindrance on lymphatic flow, thereby exacerbating RA. Thus, the depletion of local B-in cells in the joint and DLNs is therefore anticipated to confer therapeutic benefits in RA by attenuating both the inflammatory response and lymphatic dysfunction. The depletion of B-in cells using anti-CD20 mAbs in TNF-Tg mice in the onset of RA effectively prevented knee flare, while therapeutic intervention targeting collapsed DLNs ameliorated inflammatory-erosive arthritis 62, 106. Furthermore, the B cell depleting antibody Rituximab is employed as an alternative therapy for RA in clinical practice 147, 161, 162. However, progression of RA was observed in a subset of patients, while mild to moderate side effects associated with systemic Rituximab treatment were reported about 85% of patients 162. Recently, investigation has been conducted on the localized delivery of B cell depletion therapy in RA joints with the aim of mitigating off-target adverse effects. The results revealed that intra-articular injection of a B cell depletion antibody effectively enhances local exposure to DLNs of joints, thereby facilitating dose reduction and minimizing systemic toxicities in RA mice models 163, 164. Despite OA not being classified as an immune-mediated inflammatory disease within adaptive immunity, there have been documented instances of autoantibodies in synovial tissue and alterations in the characteristics of circulating and local B-cells with immunological functions 165-167. These findings provide evidence suggesting that B-cells may contribute to the pathogenesis of OA. The potential for therapeutic intervention in OA by targeting B cells, as well as the possibility of excessive B-cell activation serving as a biomarker for predicting disease progression or clinical severity, remains to be elucidated 168.

**VEGF-C/VEGFR3 treatment.** The VEGF-C/VEGFR3 signaling pathway represents a prominent target for increasing lymphangiogenesis and enhancing lymphatic drainage capacity, thereby suggesting that elevating the content of VEGF-C or selectively targeting LECs with VEGFR3 within joints may hold promising therapeutic potential in arthritis. For RA, inhibition of lymphangiogenesis and lymphatic drainage through systemic blockade of VEGFR3 exacerbates the severity of inflammation 99, whereas intra-articular administration of VEGF-C adeno-associated virus (AAV) mitigates joint damage in RA by promoting local lymphatic drainage in mice 169. For OA, synovial lymphatic drainage is impaired during OA progression. A recent study found that a decrease in the expression of VEGF-C and genes associated with the VEGFR3 signaling pathway was observed in the OA synovium of aged mice; furthermore, intra-articular injection of VEGF-C156S, a mutant form of VEGF-C that specifically binds to VEGFR3^170^, enhances synovial lymphatic drainage and attenuates tissue damage in aged mice 120. Based on these evidences, targeting VEGF-C/ VEGFR3 signaling is a promising approach for regulating lymphatic vessels in arthritis treatment; however, further investigation is required to address existing unclears. Firstly, AAV-mediated delivery of VEGF-C overcomes the short half-life of recombinant VEGF-C, and studies were reported to assess the short-term safety of this approach for potential clinical use 171, 172. Given that arthritis is a chronic disease, it is imperative to investigate the long-term effects and safety profile of AAV-based delivery of VEGF-C for joints. Additionally, the VEGF-C/VEGFR3 signaling pathway potentially modulates chondrocytes 120, macrophages 173, and bone homeostasis 31, 58, 174 in joints, necessitating further investigation.

**iNOS inhibitors.** Inflammation triggers the upregulation of iNOS, resulting in excessive production of NO, which impairs the contraction of LMCs and diminishes lymphatic drainage. Thus, inhibitors targeting iNOS may serve as potential treatments to preserve lymphatic function in joints with inflammation. For RA, preliminary findings of iNOS inhibition in animal models of acute arthritis exhibited promising outcomes 175, 176. Furthermore, local administration of L-N6-(1-iminoethyl) lysine 5-tetrazole-amide, an iNOS inhibitor, into inflamed paws of TNF-Tg mice led to restoration of lymphatic vessel contractions and improved drainage 66. Additionally, numerous plant extracts and small molecules derived from plants have demonstrated promising potential in regulating lymphatic function through the inhibition of iNOS, as evidenced by animal models of arthritis 66, 67, 177-179. For OA, the inhibition of iNOS in previous OA studies is considered to have chondrocyte-protective effects, leading to a decrease in general MMPs activity, as well as a reduction in the incidence and size of osteophytes and cartilage lesions in OA models 179. However, the impact of iNOS inhibition on lymphatic vessels remains unclear during OA progresses. Although animal studies provide support for the investigation of iNOS inhibitors as potential disease-modifying interventions for arthritis, including OA and early RA, no successful clinical trials evaluating the efficacy of these agents have been reported 180, 181. The possible explanation is that an early iNOS-independent phase followed by a subsequent iNOS-dependent phase have identified in the development of RA, suggesting that targeted inhibition of selective iNOS would yield more pronounced effects on severe RA 64, 65. Moreover, considering the irreversible nature of LMCs damage and its limited ability to fully recover after severe injury 32, 182, it is crucial to further investigate the specific timepoints at which iNOS-dependent arthritic attenuation may occur.

## 6. Perspective

In general, lymphatic vessels plays a pivotal role in maintaining joint homeostasis, dysfunction is intricately linked to inflammatory joint diseases. Further investigation is warranted to explore the dynamic changes and functional significance of lymphatic vessels at different stages of RA and OA, facilitating the identification of an optimal intervention window for clinical practice. Moreover, it is imperative to conduct a comprehensive investigation utilizing techniques such as single-cell sequencing and lineage tracing in order to thoroughly explore the key cellular subsets and molecular characteristics that underlie pathological alterations in lymphatic vessels of these diseases. The exploration of more specific molecular targets, in conjunction with tailored drug delivery modes, is crucial for optimizing therapeutic strategies and enhancing treatment efficacy efficiently. We anticipate that future advancements will yield more efficacious strategies for modulating joint lymphatic vessels, thereby enhancing the clinical outcomes of inflammatory joint diseases, such as RA and OA.

## Figures and Tables

**Table 1:** Potential therapy by targeting the function of lymphatic vessels on RA and OA

Drug		Administration	Model	Outcome	References
Anti-TNF treatment	Anti-TNF IgG1 antibody	Systemic administration 10 mg/kg/week	TNF-Tg mice	Decreased synovial and lymph node volumes without a reduction of lymphatic vessels; Restored lymphatic contractions; Potential enhancement of inflammatory cell egress; Not improved LMCs defects.	82, 98, 148, 182
	Certolizumab pegol	Systemic administration 400 mg monthly	RA patients with active flare of a single wrist or knee	Linear inverse correlation between lymph node volume and joint pain	101
	Ginsenoside Rg1	Systemic administration 20 mg/kg daily	TNF-Tg mice	Improved lymphatic drainage; Increased LMCs coverage; Reduced inflammation in LECs and bone erosion.	47, 183
	Etanercept	Local lymphatic delivery by nanotopography (SOFUSA™)	A rat model of collagen-induced arthritis	More favorable pharmacodynamics than subcutaneous or intravenous administration; Increased lymphatic pumping and reduced swelling of joints.	154
	Etanercept	Systemic administration 25-50 mg/week	Inflammatory hand OA patients	Not relieve pain; Radiographic remodeling of subchondral bone.	155
	Adalimumab	Systemic administration 40 mg/week	Inflammatory hand OA patients	Not show any effect on pain, synovitis or bone marrow lesions.	156
Proteasome inhibitors	Bortezomib	Systemic administration 0.25 mg/ml in a 5-μl	MLI-induced OA	Decreased cartilage loss; Reduced the expression of inflammatory genes by LECs; Improved lymphatic drainage.	118
B cell depletion therapy	Anti CD20 mAbs (18B12 IgG2a)	Systemic administration 10 mg/kg every 2 weeks	TNF-Tg mice	Decreased synovial volumes; Converted collapsed DLNs to expanding DLNs; Increased lymphatic clearance Without recovery of the lymphatic pulse Decreased cartilage loss.	62, 106
	Rituximab	Systemic administration 1000 mg twice	RA patients on methotrexate with resistance to TNF inhibitors	Inhibit the progression of structural joint damage; Mild to moderate side effects.	147, 161, 162
	Anti-CD20 antibody (Cy5-αCD20) Rituximab	IA compared to SC or IV administration	Collagen-induced arthritis mice Sprague-Dawley rat	IA with a greater B cell depletion in DLNs of joints.	163, 164
VEGF-C/VEGFR3 treatment	AAV-VEGF-C	IA administration	TNF-Tg mice	Increased lymphangiogenesis; Improved lymphatic drainage; Attenuated joint tissue damage.	56, 169
	VEGF-C156S	IA administration	Age-Related OA	Improved lymphatic drainage; Attenuated joint tissue damage.	120
iNOS inhibitors	Ferulic acid, L-NIL L-NAME	Local administration L-NIL (4 mg/kg) Systemic administration Ferulic acid (20 mg/kg/day) L-NIL or L-NAME (100 ng/ml in drinking water)	TNF-Tg mice	Improved lymphatic drainage; Restored lymphatic contractions; Attenuated joint tissue damage.	66
	Fang-Ji-Huang-Qi-Tang decoction	Systemic administration	Collagen-induced arthritis mice	Increased lymphangiogenesis; Improved lymphatic drainage; Attenuated joint tissue damage.	177
	Du-Huo-Ji-Sheng-Tang decoction	Systemic administration	TNF-Tg mice Zebrafish	Increased lymphangiogenesis Improved lymphatic drainage; Attenuated joint tissue damage.	178
	Total saponins of Panax notoginseng	Systemic administration	TNF-Tg mice	Prevented LMCs apoptosis; Improved lymphatic drainage; Attenuated joint tissue damage.	67
	Cindunistat (SD-6010)	Systemic administration 50 or 200 mg/day	Symptomatic knee OA patients (KLG 2 or 3)	Less joint space narrowing KLG2 patients during early stage of treatment; Not slow OA progression in KLG3 patients.	180
	GW274150	Systemic administration 60 mg/day	RA patients with DAS28 scores ≥4.0	A trend towards reduction in synovial thickness and vascularity without statistically significant.	181

**Abbreviations:** AAV: Adeno-associated virus; IA: Intra-articular; iNOS: Inducible Nitric oxide synthase; IV: intravenous; KLG: Kellgren and Lawrence Grade; L-NAME: Nω-nitro-L-arginine methyl ester; L-NIL: L-N6-(1-iminoethyl) lysine 5-tetrazole-amide; MLI: Meniscal ligamentous injury; OA: Osteoarthritis; RA: Rheumatoid arthritis; SC: Subcutaneous; TNF: Tumor necrosis factor, VEGF-C: Vascular endothelial growth factor
